# An overview of the trajectory of Brazilian individuals with 22q11.2 deletion syndrome until diagnosis

**DOI:** 10.1186/s13023-022-02225-9

**Published:** 2022-02-21

**Authors:** Isabela Mayá Wayhs Silva, Vera Lúcia Gil-da-Silva-Lopes

**Affiliations:** Department of Translational Medicine, Area of Medical Genetics and Genomic Medicine, Faculty of Medical Science, State University of Campinas (Unicamp), Tessália Vieira de Camargo Street, 126, Campinas, SP 13083-887 Brazil

**Keywords:** 22q11.2 Deletion syndrome, DiGeorge syndrome, Access to health care, Clinical management, Healthcare utilization, Multidisciplinary care age of diagnosis, Public health

## Abstract

**Background:**

22q11.2 deletion syndrome (22q11.2DS) is a rare disease with an important characteristic—clinical heterogeneity. The diversity of organs, regions, and systems of the body that can be affected requires periodic updating of health professionals so that they can recognize these clinical signs as belonging to 22q11.2DS. Updated professionals are equally important for the appropriate and timely clinical management of individuals with a positive diagnosis. In this context, this article aimed to map and analyze the access to healthcare for individuals with 22q11.2DS until the moment of diagnosis.

**Results:**

We analyzed the clinical data of 111 individuals with 22q11.2DS registered in the Brazilian Database on Craniofacial Anomalies (BDCA) from 2008 to 2020. In this study, individuals were diagnosed at a median age of 9 years (mean = 9.7 years). Before the genetic investigation, they accessed 68.75% of the internationally recommended evaluations available at BDCA. Recurrent 22q11.2DS clinical manifestations such as delayed neuropsychomotor development, lip and/or palate defects, cardiac malformation and/or hematological/immunological alteration co-occurred in at least 72.06% of individuals. Cardiac malformation was the only clinical alteration that lowered the median diagnostic age, corresponding to 6.5 years of age with a cardiac malformation versus 11 years of age without one (p = 0.0006).

**Conclusions:**

In Brazil, 22q11.2 DS is under-recognized, and early diagnosis and management of affected individuals are still a distant reality. In this sense, 22q11.2 DS suspicion followed by the elimination of obstacles for its diagnosis confirmation is essential to increase life expectancy and improve the quality of life of these individuals in Brazil.

## Background

The 22q11.2 deletion syndrome (22q11.2DS) is a rare genetic disease. It has an estimated incidence among different populations of 1:3000 to 1:6000 among live births, which ranks it as the most frequent microdeletion in humans [1, 2].

The first and most frequent clinical manifestations in 22q11.2DS are the dysmorphic features, hypocalcemia, and/or hypoparathyroidism, neuropsychomotor developmental delay (NPMDD) and heart and lip/palate defects. From the first year of life until preadolescence, delayed speech acquisition or hypernasal voice and behavioral alterations such as anxiety, attention deficit, hyperactivity, socialization problems and learning difficulties are also common. During adolescence and adulthood, neuropsychiatric disorders such as bipolar disorder, schizophrenia, anxiety and intellectual disability are recurrent and can interfere or limit their social integration [3–5].

Although there are more common alterations in each age group, studies associated over 180 clinical manifestations with 22q11.2DS [2, 6]. Clinical heterogeneity makes it difficult to appreciate the insidious signs and symptoms as belonging to a single phenotype, increasing the complexity of the suspicion and diagnosis of 22q11.2DS. In Brazil, 10 years is the average diagnosis age for individuals with this syndrome [7, 8].

The management of this condition should be personalized, based on the clinical changes and severity of the symptoms observed. To handle the planning of this management, there are guidelines for clinical and laboratory investigations for the diagnosis and post-diagnosis follow-up in the different age groups [3].

The assessments recommended to establish individual health care and management include pediatric/clinical, cardiac, nasopharyngeal, immunological, hematological, endocrinological evaluations, renal, hearing, ophthalmic, orthopedic, dental, psychopedagogical, psychiatric and genetic counseling. Gynecological evaluation, sex education and genetic orientation of the proband in adolescence and adulthood add to the list [4, 9].

In Brazil, the Comprehensive Care Policy for People with Rare Diseases (PAIPDR—Ordinance GM/MS no 199/2014) [10], within the Unified Health System (SUS), regulates the treatment of individuals with rare diseases. This system currently has 17 enabled reference centers [11]. Therefore, considering the Brazilian population, the territorial extension and difficulty of suspecting 22q11.2DS, there are obstacles to access confirmatory tests, genetic orientation and clinical management [12]. Within this framework, the notification of rare diseases is not compulsory in the country [13], making a health situation analysis of the population with 22q11.2DS impossible, based on data such as prevalence, clinical management, health needs and life expectancy. Additionally, no guidelines are defined by the Ministry of Health for the healthcare of this population group.

To improve healthcare for individuals with craniofacial alterations through the production of scientific evidence, Brazil's Craniofacial Project (BCFP) was established. It is a voluntary, multicenter, inter-institutional initiative [14]. Since 2006, BCFP has developed strategies to help the diagnosis of 22q11.2DS, such as refinement of the common phenotype and registration of clinical follow-ups in the BDCA. In this context, the BCFP has brought information to subsidize scientific approaches such as this one, which leads to the recognition of the health needs of this population group [8, 15]. Furthermore, BCFP has drawn up a 22q11.2DS clinical management guide in Portuguese [5].

The investment in training health professionals and continuous and proper clinical management, made possible by early 22q11.2 DS diagnosis, potentiates the reduction of morbidity and mortality, increase in life expectancy, and reduction of treatment expenses and helps caregivers prepare for the natural evolution of the syndrome [16–18].

Based on the information recorded in the BDCA on individuals with 22q11.2DS, the present study aimed to characterize the clinical investigation carried out until the diagnostic conclusion and identify the factors interfering in this investigation. The knowledge of this trajectory allows recognizing and subsidizing the enhancement and targeting of public policies to improve the suspicion of 22q11.2DS and the access to adequate health management.

## Methods

The Ethics Committee of the State University of Campinas approved this study (CAAE: 2477419.1.0000.5404), and all participants or their guardians signed the informed consent form.

We analyzed the primary data of 111 individuals with 22q11.2DS, from BDCA, linked to BCFP [14, 19]. This data was collected from 2008 to 2020.

### Diagnosis of 22q11.2DS

All individuals participating in this study presented positive results for typical deletion in region q11.2 of chromosome 22. BCFP performed diagnostic examinations of all cases as part of research without cost to the family. The diagnostic methods used were FISH and/or MLPA and CMA, according to the research projects through the years. The age of diagnosis ranged from 0 to 33 years and is equivalent to the time when genetic research was accessed. Individuals diagnosed at less than 1 year of age had their age of diagnosis classified as 0.

### Clinical aspects and health interventions

The time of diagnostic test access is equivalent to the registration time in BDCA and the time of collection of data on clinical aspects and health interventions. Therefore, the age of diagnosis is the same as that when the parents were genetically counseled.

The international recommendations for clinical management at the time of diagnosis of 22q11.2DS include assessment of the following aspects: (a) cardiac, (b) nasopharyngeal, (c) hearing; (d) psychiatric; (e) hematological/immunological, (f) endocrinological, (g) ophthalmic, (h) renal, (i) orthopedic, (j) genetic counseling, (k) pediatric/ clinical evaluation and l) assessment of social inclusion [4, 9].

Out of these assessments, the ones available in the BDCA to analyze the therapeutic itinerary until the diagnosis were as follows: (a) cardiac, (b) nasopharyngeal, (c) hearing, (d) psychiatric, (e) hematological/immunological, (f) endocrinological, (g) ophthalmic and (h) renal. Psychopedagogical assessment was also available in the BDCA and analyzed separately as a supportive therapy. Consequently, it had not been used to map the therapeutic itinerary.

The clinical findings associated with 22q11.2DS and available for analysis in BDCA were neuropsychomotor development, lip and/or palate, cardiac, psychiatric, hematologic/immunological, endocrinological, auditory, ophthalmological, genitourinary and gastrointestinal tract. Clinical analysis include a different total sample for each clinical finding due to variations on available records in BDCA.

### Statistical analysis

Frequency tables for categorical variables and measures of position and dispersion for numerical variables were used to describe the sample profile. We used chi-square or Fisher’s exact test to compare categorical variables and Mann–Whitney and Kruskal–Wallis tests to compare numerical measurements. The significance level adopted was 5%.

## Results

### General data of the casuistry and age at diagnosis

Of the 111 individuals, 64/111 (57.7%) were female and 47/111 (42.3%) were male. The median age of the sample group was 9 years of age (sd = 7.2, mean = 9.7 years, mode = 6). The geographical distribution shows that 6/111 (5.4%) come from the Northeast, 39/111 (35. 1%) from the Southeast and 66/111 (59.5%) from the South. The median age at diagnosis was 9 years, with a minimum value of 0 years and a maximum of 33 years (sd = 7.2, mean = 9.7 years, mode = 6).

### Clinical changes and age of diagnosis

The five most recurrent clinical alterations in the total sample (N = 111) were NPMDD 85/97 (87.6%), lip and/or palate defects 87/101 (86.1%), cardiac malformation 68/96 (70.8%), hematological/immunological alteration 57/81 (70.4%) and psychiatric disorder 49/83 (59%) (Table 1).

**Table 1:** Clinical manifestations in the total sample

	No of individuals/total	%
NPMDD*	85/97	87.63
Lip-palatal defect	87/101	86.14
Cardiac Malformation	68/96	70.83
Hematological/immunological alteration	57/81	70.37
Psychiatric disorder	49/83	59.04
Alteration of the gastrointestinal tract	43/79	54.43
Hearing deficiency	36/81	44.44
Ophthalmological alteration	30/71	42.25
Endocrinological alteration	13/35	37.14
Alteration of the genitourinary tract	12/60	20

^*^Neuropsychomotor developmental delay

Individuals with a cardiac malformation had a lower median diagnostic age compared to individuals without one, corresponding to 6.5 and 11 years of age (p = 0.0006242), respectively. Conversely, individuals with psychiatric disorders and individuals with lip and/or palate defects had a higher median diagnostic age than that found in individuals without these alterations, equivalent to 10 versus 7.5 years (*p* = 0.05677) and 10 versus 1.1 years (*p* = 0.0004966), respectively.

### Concomitant clinical manifestations

Among the 85 individuals with NPMDD, 74 (87.1%) also presented lip and/or palate defect, 49 (57.7%) a cardiac malformation and 45 (52.9%) a hematological/immunological alteration.

In the group of 87 individuals with lip and/or palate defects, 74 (85.1%) had NPMDD, 47 (54%) a cardiac malformation and 44 (50.6%) a hematological/immunological alteration.

In the 68 individuals with a cardiac malformation, 49 (72.1%) presented with NPMD, 47 (69.1%) a lip and/or palate defect and 33 (48.5%) a hematological/immunological alteration.Among the 57 individuals with a hematological/immunological alteration, 45 (79%) also presented NPMDD, 44 (77.2%) with lip and/or palate defects and 33 (57.9%) with a cardiac malformation.

### Access to expert assessment and age of diagnosis

Among the 12 evaluations recommended at the time of diagnosis, eight were available in the BDCA. The average access was 5.5 (68.75%) evaluations per individual (minimum value equal to 1 and a maximum of 8, median and mode = 6).

The three most accessed evaluations were nasopharyngeal 107/111 (96.4%), cardiac 104/111 (93.7%), and psychiatric 83/111 (74.8%). The least accessed was endocrinological 35/111 (31.5%) (Table 2). Individuals with ophthalmological, psychiatric and nasopharyngeal assessments had a higher median age of diagnosis, equivalent to 10 versus 7 years (*p* = 0.004917), 10 versus 4 years (*p* = 0.002412) and 9 versus 0 years (*p* = 0.002302), respectively.

**Table 2:** Health assessments available at BDCA and performed by the total sample

	No of individuals/total = 111	%
Nasopharyngeal	107	96.40
Cardiac	104	93.69
Psychiatric	83	74.77
Hearing	81	72.97
Hematological/immunological	81	72.97
Ophthalmological	71	63.96
Renal	48	43.24
Endocrinological	35	31.53

### Access to supportive therapy

Among the 111 individuals, 14 (12.6%) accessed psychopedagogical assessment.

## Discussion

Although 22q11.2DS is the most frequent microdeletion in humans, its diagnosis and management are still universally challenging [3, 4, 20, 21]. Considering the continental dimensions of Brazil and the key access to health for the population being through SUS [22], it is essential to seek strategies that facilitate suspicion, investigation and management of this clinical condition. The perspective of this study is to characterize the clinical trajectories of individuals registered in BDCA until their 22q11.2DS diagnosis. The difference in the percentage of cases between Brazilian regions presented herein reflects only the demand registered by the BCFP participating centers and has no epidemiological value.

Individuals with important characteristics of 22q11.2DS are arriving late to the reference services, which may compromise therapeutic interventions and genetic counseling of parents. In Canada, the mean age of diagnosis of 22q11.2DS is 4.7 years [20], and adults with 22q11.2DS and continuous interdisciplinary follow-up have a life expectancy of 46.4 years [23].

In the sample described herein, individuals with a psychiatric disorder (49/83) had a higher median age of 22q11.2DS diagnosis (10 years with psychiatric disorder versus 7.5 years without one). Up to 40% of individuals with intellectual disability have a psychiatric disorder [24], which can contribute to the deficient association between this phenotype and 22q11.2DS. In 22q11.2DS, these alterations manifest more frequently in adolescence [4, 5, 25], which would make the early diagnosis impossible if this was the most evident characteristic of this syndrome in an individual. Instead, in this study, over 53.1% of individuals with a psychiatric disorder also presented other important characteristics of 22q11.2DS, such as NPMDD, lip and/or palate defects, cardiac malformation and/or hematological/immunological alteration.

Similarly, individuals with lip and/or palate defects alone had a higher median age of diagnosis (10 years versus 1.1 years without lip and/or palate defects). These defects occur more frequently in isolation [26] but are present in at least 600 other Mendelian syndromes [27]. Among these, 22q11.2DS is the most frequent microdeletion [7, 28]. In this study, at least 50.6% of the individuals who presented lip and/or palate defects (87/101) also presented other clinical manifestations of 22q11.2DS, such as NPMDD, cardiac malformation and/or hematological/immunological alteration.

In total, 70.8% (68/96) of the individuals had a cardiac malformation—one of the most recognized characteristics of 22q11.2DS. The presence of a cardiac malformation was the only significant variable that reduced the diagnostic age (median of 6.5 years with a cardiac malformation versus 11 years without one). Other studies had similar results [20, 29].

In our study, at least 72.06% of the individuals present concomitantly two or more recurrent clinical manifestations indicative of 22q11.2DS. These results suggest difficulty in clinically suspecting 22q11.2DS even with the most common features. The diagnosis in individuals with nonspecific findings of 22q11.2DS such as intellectual disability or hypernasal voice is even more challenging. Obstacles in referral and access to the genetic service may also delay diagnosis. In any case, the need for improvement in the flow of primary care-specialized/reference service is a reality. In this sense, Monteiro and collaborators proposed some clinical characteristics for suspecting 22q11.2DS and laboratory investigation [15], which were later validated [8].

Using this data to produce information resources aimed at training health professionals is essential for better effectiveness and efficiency of the flow of primary care-reference services. This information set can also contribute to the establishment of a national protocol for clinical management of 22q11.2DS and the national standardization of care. Finally, both strategies are important tools to achieve early diagnosis of 22q11.2DS [8].

Still, within PAIPDR, individuals referred to the reference services in rare diseases should return to primary and secondary care to receive multi-professional care according to the therapeutic plan established by the reference team [10]. Counter-reference enables individuals to perform evaluations in their regional health units, increasing treatment adherence. In this study, individuals accessed 68.75% of the evaluations available in the BDCA and recommended at the time of 22q11.2DS diagnosis [9]. These evaluations happened before the diagnosis, reinforcing health professionals’ unfamiliarity with this syndrome and (or) the difficulty to access genetic evaluation and tests.

Individuals that accessed psychiatric and individuals with nasopharyngeal assessments had a higher median age of diagnosis, of 10 versus 4 years of age and 9 versus 0 years, respectively. These results add to those regarding the age of diagnosis with psychiatric and lip and/or palate defects and corroborate the perception of obstacle(s) in the access to the genetics service.

Endocrinology was the least accessed specialty, mentioned by 31.5% (35/111) of the sample group. In 22q11.2DS, the most recurrent endocrinological alteration is idiopathic hypocalcemia, which may present in up to 60% of individuals since the neonatal period [5]. This is a classical 22q11.2DS manifestation and therefore important to suspect clinically and refer to the medical genetics service. We should note that the number of endocrinologists working in SUS is 6990 [30], which makes universal access to this professional very restrictive.

Only 12.6% of the sample group accessed psychopedagogists. Psychopedagogical follow-up is a key aspect for recognition of individual potential and difficulties in school performance and psychosocial insertion. Psychopedagogy is among the support therapies advocated in reference centers for rare diseases and is mainly indicated in the presence of NPMDD [10]. In this study, NPMDD was the recurrent clinical alteration. The number of psychopedagogists working in SUS is 1479 [31], which makes universal access to this professional difficult.

Given the diversity and quantity of rare diseases, suspecting this specific condition is challenging, but there are possible paths to tread when faced with a complex clinical disease without a defined cause.

The recording of the medical history is up to primary healthcare, which includes a meticulous clinical evaluation, active listening, recording of family history and anamnesis. The referral to specialized care allows the performance of a complete check-up, facilitating the detection of cardiac, immunological, endocrinological, neurological, genitourinary and gastrointestinal tract alterations [10]. This information set can narrow the range of possible rare diseases and contain possible health aggravations in individuals who have not yet accessed the reference service for rare diseases.

Data on the post-diagnosis management and life expectancy of Brazilians with 22q11.2DS are not available. Its absence makes it impossible to optimize the PAIPDR to ensure increased life expectancy with early diagnosis and increased quality of life. The consequences of delayed diagnosis associated with incomplete management are multitudinous. We highlight the worsening of untreated clinical manifestations and in the prognosis of this individual, the delay in the family’s preparation to deal with the evolution of the clinical condition and the increase in the health costs for both the patient and the state.

The lack of 22q11.2DS data from the North and Midwest and the few cases from the Northeast prevented the comparison of the therapeutic itinerary until the moment of diagnosis between the different regions of Brazil. It is noteworthy that BCFP does not have partner centers in the North and Midwest. However, considering that the socioeconomic and demographic differences between the five regions of the country correlate directly with the availability and quality of health services [32, 33], the number of evaluations accessed by individuals with 22q11.2DS from different regions may vary.

From this perspective, the small proportion of genetic services in the North [11, 34] with the reduced number of geneticists in Brazil, allocated mostly in the Southeast [35, 36], makes fair access to early diagnosis less probable.

**Fig. 1:**
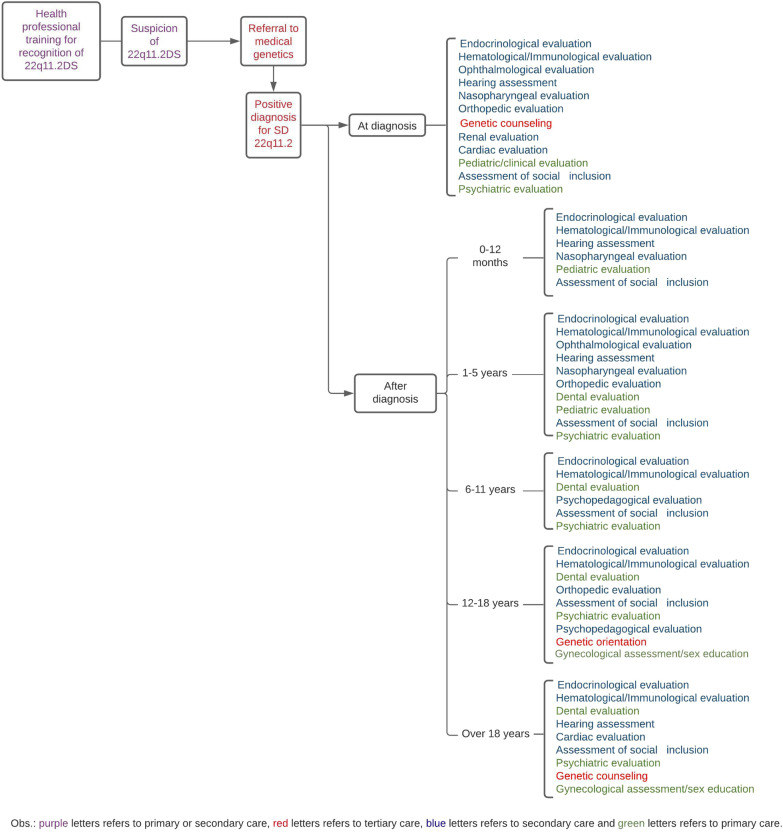
Flowchart for clinical management of 22q11.2DS at the Brazilian Unified Health System

Following the international recommendations for diagnosis, clinical management of 22q11.2DS and the characteristics of the Brazilian Unified Health System [4, 9] we suggest a flowchart for the general line of health care (Fig. 1).

Whereas clinical management should be based on recommendations for each age group, the establishment of the therapeutic plan should be individualized. The proposed flowchart may guide the multidisciplinary team to define the longitudinal therapeutic plan. If this strategy is established as public health policy, the integral approach towards each individual will favor its complete biopsychosocial insertion.

This was a retrospective, cross*-*sectional study and, therefore, it has restrictions on working with available data collected through the years. Furthermore, some information is collected from the report of individuals with 22q11.2 DS and/or their parents or legal guardians; therefore, it may contain a comprehension and memory bias*. C*onsidering Brazil’s inequalities regarding socioeconomic aspects and access to health care, it also may contain biases associated with availability and ease of access to health services.

## Conclusions

In conclusion, even in the presence of recurrent clinical manifestations co-occurring in people with 22q11.2 DS, its diagnosis in Brazil is delayed. Additionally, people with 22q11.2DS also have fewer assessments than that available from the BDCA and possibly even less than internationally recommended. Therefore, there is a clear link between early suspicion and diagnosis of 22q11.2DS, the training of health professionals and access to diagnostic tests. From the diagnosis onwards, the structuring of clinical management according to specific lines of care would lead to timely interventions to obtain the best results for each clinical situation.

## Data Availability

Not applicable.

## Abbreviations

22q11.2DS: 22Q11.2 Deletion Syndrome
NPMDD: Delayed neuropsychomotor development
BCFP: Brazil’s CranioFacial Project
BDCA: Brazilian Database on Craniofacial Anomalies
PAIPDR: Comprehensive Care Policy for People with Rare Diseases
SUS: Unified Health Care System
CMA: Chromosomal Microarray Analysis
FISH: Fluorescent in situ Hybridization
MCA: Multiple Congenital Anomalies
MLPA: Multiplex Ligation Probe-dependent Amplification
Famerp: Faculty of Medicine of São José do Rio Preto
UFRGS: Federal University of Rio Grande do Sul
CAIF: Cleft Lip and Palate Integral Care Center
CADEFI: Center for Attention to Defects of Face
HUPAA: Professor Alberto Antunes Hospital
UFAL: Federal University of Alagoas
APAE: Association of Parents and Friends of the Exceptional from Sao Paulo
CAISM: Center for Comprehensive Women's Health Care
Unicamp: State University of Campinas
